# Activation and Desensitization of Peripheral Muscle and Neuronal Nicotinic Acetylcholine Receptors by Selected, Naturally-Occurring Pyridine Alkaloids

**DOI:** 10.3390/toxins8070204

**Published:** 2016-07-04

**Authors:** Benedict T. Green, Stephen T. Lee, Kevin D. Welch, Daniel Cook, William R. Kem

**Affiliations:** 1 Poisonous Plant Research Laboratory, Agricultural Research Service, United States Department of Agriculture, 1150 E. 1400 N., Logan, UT 84341, USA; stephen.lee@ars.usda.gov (S.T.L.); kevin.welch@ars.usda.gov (K.D.W.); daniel.cook@ars.usda.gov (D.C.); 2 Department of Pharmacology and Therapeutics, College of Medicine, University of Florida, 1200 Newell Road, Gainesville, FL 32610-0267, USA; wrkem@ufl.edu

**Keywords:** anabaseine, anabasine, epibatidine, nicotinic acetylcholine receptor, nicotine, pyridine alkaloid

## Abstract

Teratogenic alkaloids can cause developmental defects due to the inhibition of fetal movement that results from desensitization of fetal muscle-type nicotinic acetylcholine receptors (nAChRs). We investigated the ability of two known teratogens, the piperidinyl-pyridine anabasine and its 1,2-dehydropiperidinyl analog anabaseine, to activate and desensitize peripheral nAChRs expressed in TE-671 and SH-SY5Y cells. Activation-concentration response curves for each alkaloid were obtained in the same multi-well plate. To measure rapid desensitization, cells were first exposed to five potentially-desensitizing concentrations of each alkaloid in log_10_ molar increments from 10 nM to 100 µM and then to a fixed concentration of acetylcholine (ACh), which alone produces near-maximal activation. The fifty percent desensitization concentration (DC_50_) was calculated from the alkaloid concentration-ACh response curve. Agonist fast desensitization potency was predicted by the agonist potency measured in the initial response. Anabaseine was a more potent desensitizer than anabasine. Relative to anabaseine, nicotine was more potent to autonomic nAChRs, but less potent to the fetal neuromuscular nAChRs. Our experiments have demonstrated that anabaseine is more effective at desensitizing fetal muscle-type nAChRs than anabasine or nicotine and, thus, it is predicted to be more teratogenic.

## 1. Introduction

In grazing livestock, the in utero exposure to certain piperidinyl-pyridine alkaloid plant toxins from *Lupinus*, *Conium*, and *Nicotiana* spp. can cause developmental defects (terata) by inhibiting fetal movement [1]. These defects (arthrogyrposis, kyposis, lordosis, scoliosis, torticollis, and cleft palate) are termed multiple congenital contracture-type (MCC-type) deformities [2]. The mechanism by which these plant toxins inhibit fetal movement has been postulated to involve activation, and then desensitization, of fetal muscle-type nicotinic acetylcholine receptors (nAChRs) that are expressed by the developing fetus [1]. This mechanism is similar to the mechanism of neuromuscular block caused by depolarizing muscle relaxants, like succinylcholine [3]. These alkaloids from *Lupinus*, *Conium*, and *Nicotiana* spp. cause crooked calf syndrome in extensively-grazed cattle in the Western United States. Crooked calf syndrome negatively impacts the welfare of the calf and creates an economic burden due to increased management and veterinary costs. 

**Figure 1:**
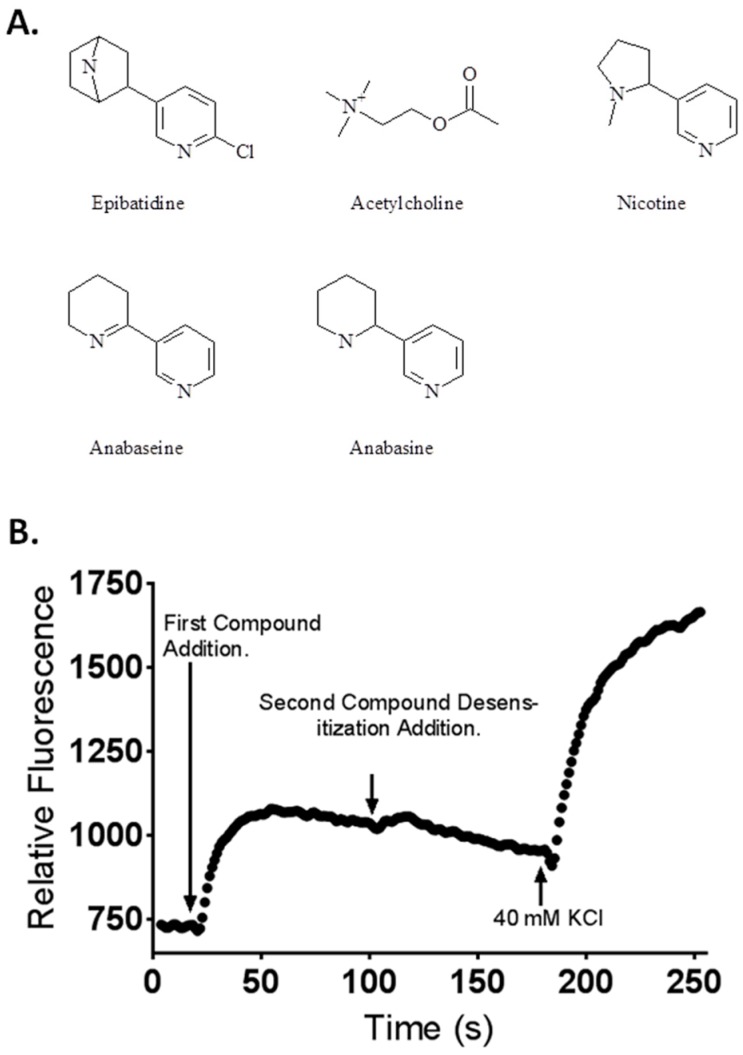
Chemical structures of compounds tested in this research (**A**). Illustration of experimental design (**B**), depicting compound additions one (measuring agonist potency and causing desensitization) and two (measuring desensitization), and the KCl addition that served as a depolarizing calibrant. The second compound addition was ACh at 10 µM (SH-SY5Y cells) or 1 µM (TE-671 cells).The change in relative fluorescence of the dye-loaded cells was detected using a Flexstation III multi-mode microplate reader.

Nicotinic acetylcholine receptors are ligand-gated cation channels and members of the cys-loop receptor family. Each receptor is a pentameric complex containing five homologous subunits. There are 17 distinct nAChR subunits; α_1–10_, β_1–4_, γ, δ, and ε [4,5] that are expressed in vertebrates. More than ten different subunit combinations have been reported to occur in mammals. In each receptor at least two C-loop-containing α subunits are present; they provide most of the two acetylcholine binding sites that must be occupied by agonists for maximal activation. All nAChRs can exist in multiple conformation states, including resting (closed channel), activated (open channel), and desensitized (agonist occupied but closed channel) states, all of which display different allosterically-linked affinities for agonists and antagonists [6,7]. Transitions between the various receptor states depend, to some degree, upon the chemical structure of the agonist. For example, the excitatory neurotransmitter acetylcholine (ACh) is more potent at activating fetal muscle-type nAChR expressed by TE-671 cells than the pyridine alkaloid nicotine, with EC_50_ values 0.3 and 17.6 µM, respectively [8]. Prolonged exposure of TE-671 cells to ACh also produces a more profound desensitization than nicotine [9]. Differences in agonist actions at the muscle receptor have been partially explained by differences in the types of non-covalent bonding interactions between agonists and the different nAChRs that are related to differences in the chemical structures of the agonists (Figure 1A) [10,11]. 

In this research, we investigated four nAChR agonists (epibatidine, the most potent known nAChR agonist, nicotine, anabasine, and anabaseine) for their ability to activate and then rapidly desensitize nAChRs. The rapid type of desensitization examined in this study has been termed “classical desensitization” and develops on a second time scale at the agonist concentrations used in our investigation [7]. We chose to examine classical desensitization based on our previously reported research of plant toxin-induced inhibition of fetal movement in goats, which has documented the nearly immediate abolition of fetal goat movement by several pyridine alkaloids, but not nicotine [12,13]. In this study we used two cell lines with fetal characteristics as models to study receptor desensitization of the two peripheral nAChRs: TE-671 cells expressing fetal human muscle-type nAChR (α1_2_β1γδ) and SH-SY5Y cells derived from embryonic central nervous system tissue expressing markers characteristic of immature catecholaminergic neurons, including predominantly autonomic-type nAChRs containing α3 and β4 subunits [14,15,16,17]. In our experiments, we used a dual agonist addition protocol, the first agonist to desensitize nAChR (see Figure 1B for a representative tracing from a typical experiment depicting initial agonist stimulation), and a second ACh addition to monitor the degree of desensitization. We report the relative abilities of four pyridine alkaloids (the plant toxins nicotine and anabasine, and the animal toxins epibatidine and anabaseine) to activate and rapidly desensitize the two peripheral nervous system nAChRs that have been implicated in MCC-type defect formation in fetal livestock and crooked calf syndrome in grazing livestock.

## 2. Results

**Figure 2:**
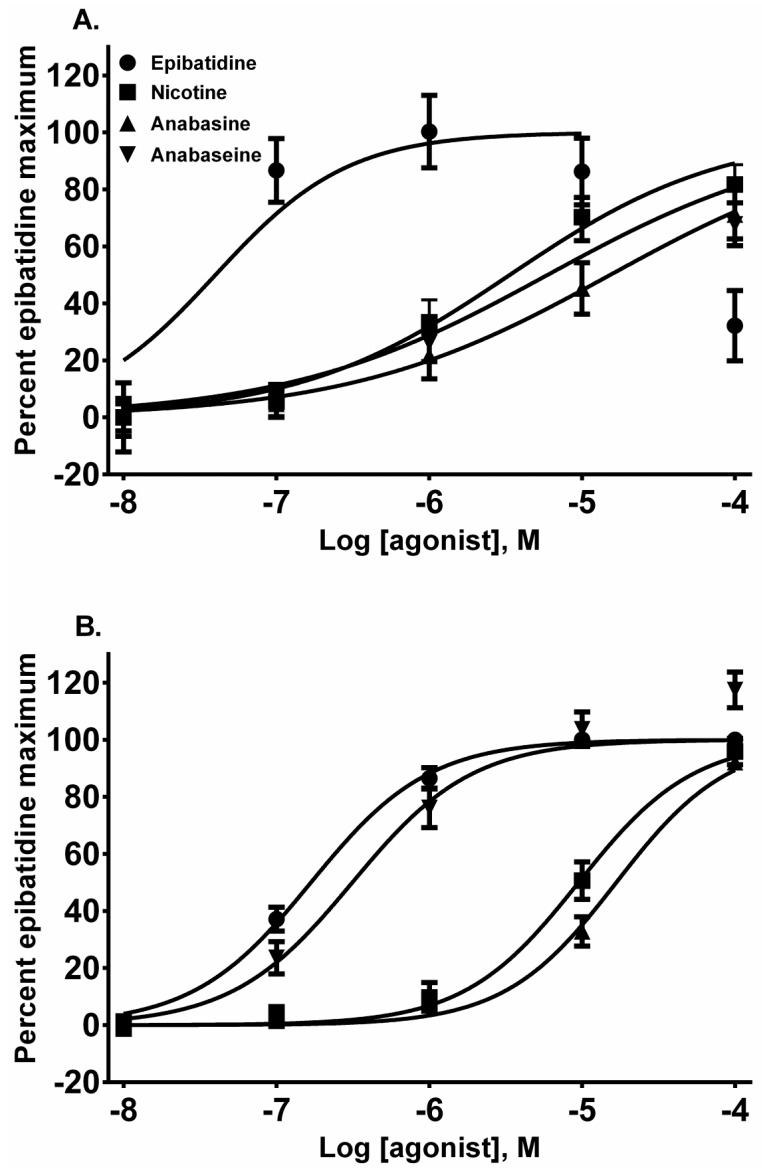
Concentration-response relationships for the four alkaloids using membrane potential sensing dye fluorescence in SH-SY5Y (**A**) and TE-671 (**B**) cells. The cellular responses to agonist were normalized to the maximum epibatidine response for each cell line. The activation concentration-response curves are the same curves as in subsequent figures.

Each alkaloid acted as a full nAChR agonist, as indicated by the near maximal membrane potential sensing dye fluorescence for each alkaloid in the activation-concentration curves for each cell line (Figure 2, EC_50_ values presented in Table 1). Interestingly, in the TE-671 cells, anabaseine was nearly as potent an agonist as epibatidine.

**Figure 3:**
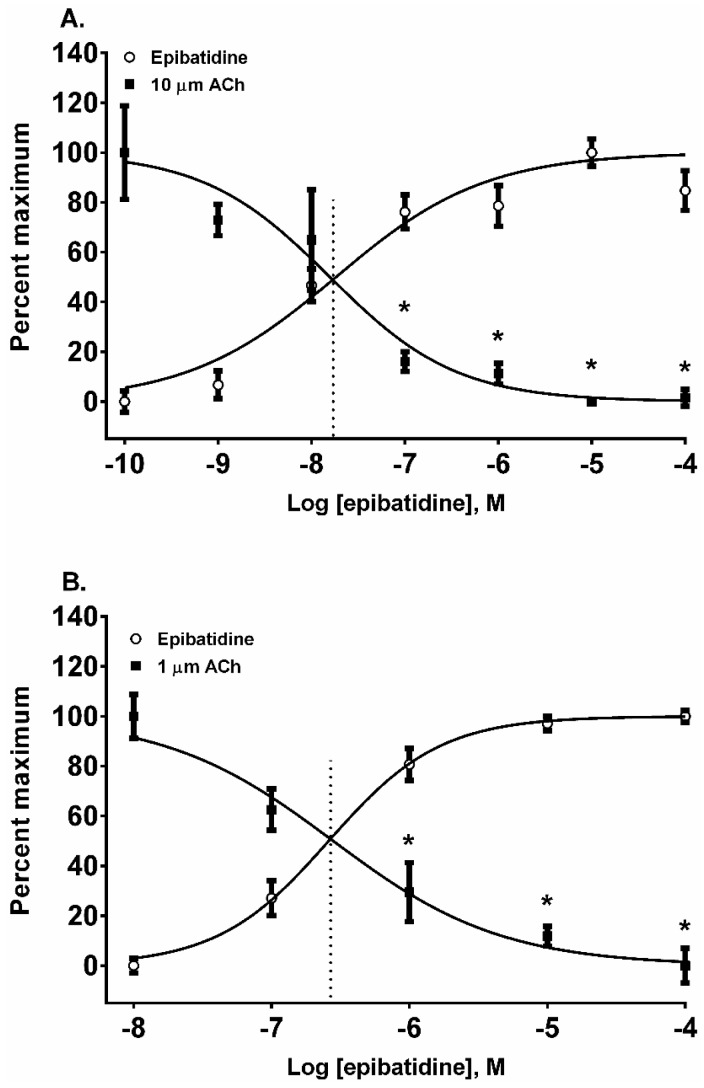
The actions of epibatidine on the activation and desensitization of nAChR expressed by SH-SY5Y (**A**) and TE-671 (**B**) cells. The membrane potential sensing dye fluorescence resulting from the addition of an agonist in log_10_ molar concentrations was measured and displayed as a percentage of the maximal agonist response. Each epibatidine and ACh datum point represents six to 10 experiments of duplicate wells for SH-SY5Y cells and four experiments of duplicate wells for TE-671 cells. * *p* < 0.05, epibatidine response versus the cellular response to a fixed concentration of ACh. The vertical dotted lines in each panel are a graphical representation of the A/D intercepts reported in Table 1.

**Figure 4:**
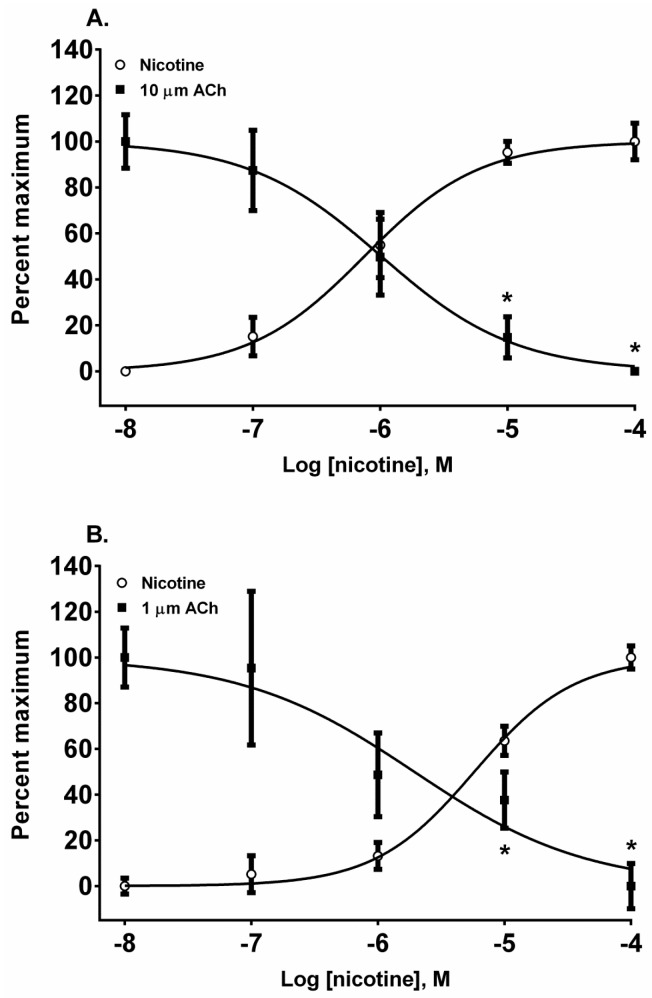
The actions of nicotine on the activation and desensitization of nAChR expressed by SH-SY5Y (**A**) and TE-671 (**B**) cells. The membrane potential sensing dye fluorescence resulting from the addition of an agonist in log_10_ molar concentrations was measured and displayed as a percentage of the maximal agonist response. Each nicotine and ACh datum point represents four experiments of duplicate wells. * *p* < 0.05, nicotine response versus the cellular response to a fixed concentration of ACh.

**Figure 5:**
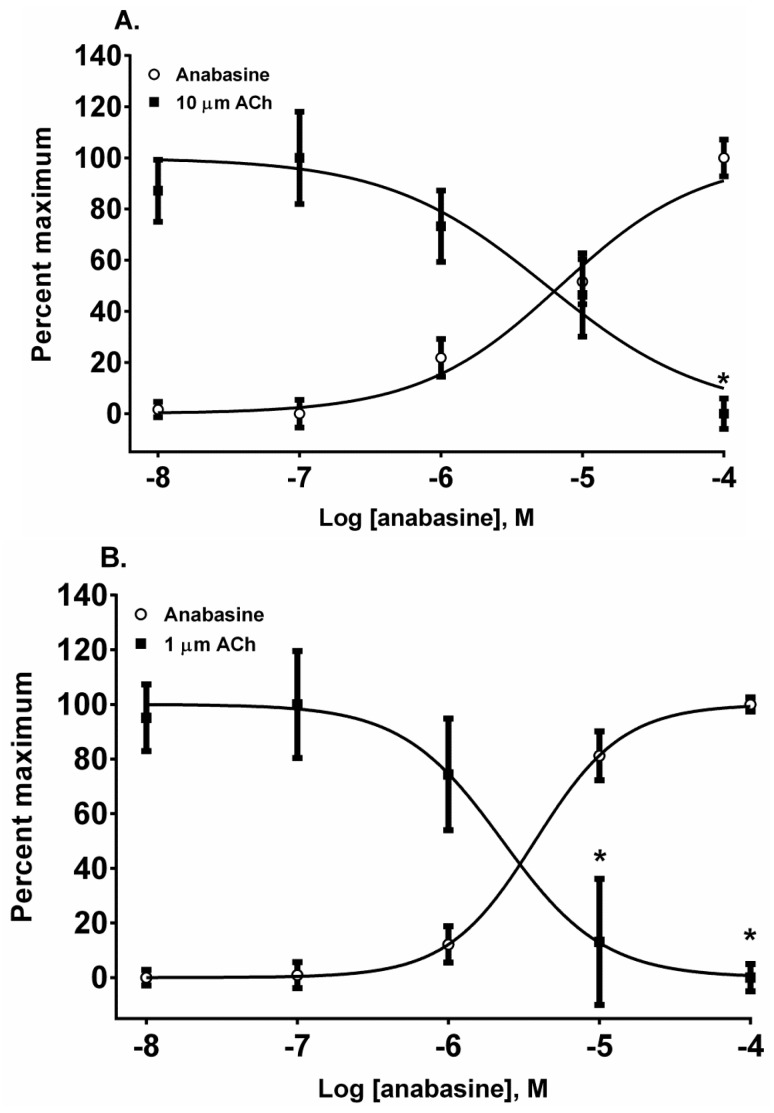
The actions of anabasine on the activation and desensitization of nAChR expressed by SH-SY5Y (**A**) and TE-671 (**B**) cells. The membrane potential sensing dye fluorescence resulting from the addition of anabasine in log_10_ molar concentrations was measured and displayed as a percentage of the maximal epibatidine response. Each anabasine and ACh datum point represents four experiments of duplicate wells. * *p* < 0.05, anabasine response versus the cellular response to a fixed concentration of ACh.

**Figure 6:**
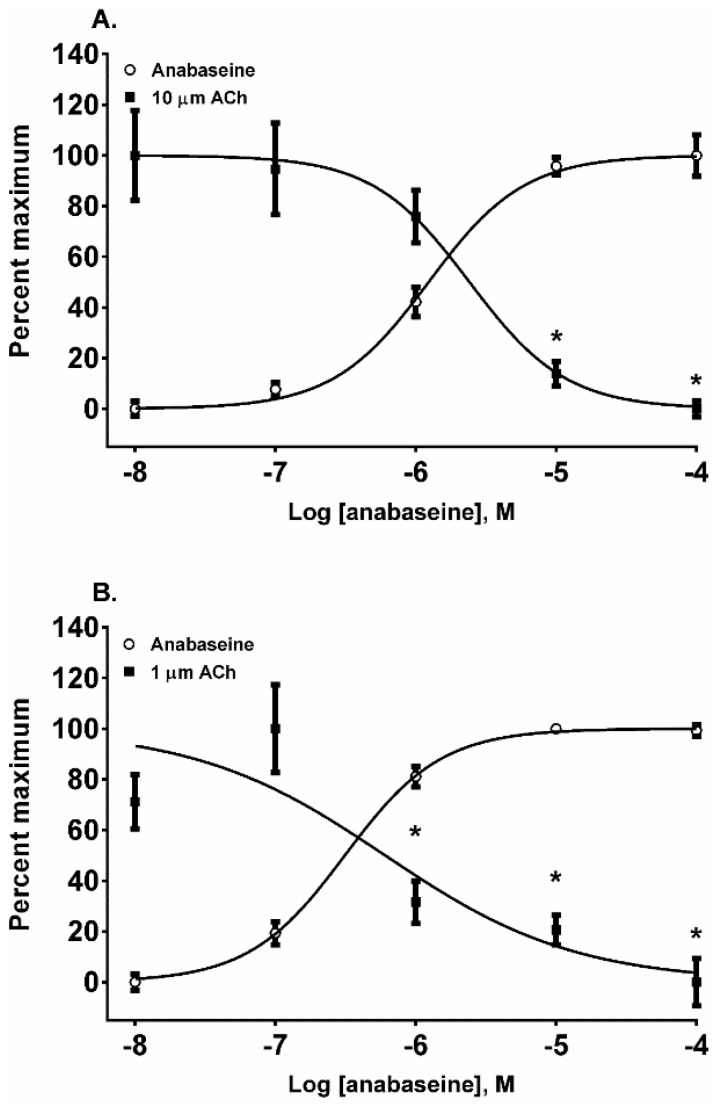
The actions of anabaseine on the activation and desensitization of nAChR expressed by SH-SY5Y (**A**) and TE-671 (**B**) cells. The membrane potential sensing dye fluorescence resulting from the addition of anabaseine in log_10_ molar concentrations was measured and displayed as a percentage of the maximal epibatidine response. Each anabaseine and ACh datum point at each anabaseine concentration represents four experiments of duplicate wells with the exception of the 0.1 µM ACh datum point in TE-671 cells which represents three experiments of duplicate wells. * *p* < 0.05, anabasine response versus the cellular response to a fixed concentration of ACh.

In addition to nAChR activation by the four alkaloids, we also assessed their ability to desensitize autonomic and neuromuscular nAChR subtypes expressed by SH-SY5Y and TE-671 cells, respectively. The nAChRs were classically desensitized by exposing the cells to five concentrations of each alkaloid in log_10_ molar increments from 10 nM to 100 µM. The resulting change in the membrane potential sensing dye fluorescence was measured and then the cells were exposed to a fixed concentration of ACh (10 µM for SH-SY5Y cells and 1 µM for TE-671 cells) to assess receptor desensitization. All of the four alkaloids were effective at desensitizing the responses to ACh in both cell lines (Figure 3, Figure 4, Figure 5 and Figure 6, calculated EC_50_, DC_50_, and activation/desensitization versus concentration curve intercept values are presented in Table 1).

Epibatidine was the most potent agonist that was tested (Figure 2A,B). It was also the most potent alkaloid for desensitizing the ACh response (Figure 3A,B). For example, the 10 µM ACh addition in SH-SY5Y cells was desensitized at 0.1, 1, 10, and 100 µM (*p* < 0.05, two-way ANOVA, Holm-Sidak’s multiple comparisons test). Similarly, the ACh additions in TE-671 cells were desensitized at 1, 10, and 100 µM (*p* < 0.05, two-way ANOVA, Holm-Sidak’s multiple comparisons test). Moreover, the A/D intercept between epibatidine and ACh in both cell lines were the smallest values calculated for any alkaloid reflecting its potency as an agonist (Table 1). 

The pyridine alkaloid nicotine does not cause MCC-type defects because it does not completely inhibit fetal movement in livestock. It was a potent nAChR agonist in both cell lines (Figure 2A,B). In SH-SY5Y cells which express a neuronal-type nAChR, the ACh response was desensitized at 10 and 100 µM by treatment with 10 µM ACh (Figure 4A) (*p* < 0.05, two-way ANOVA, Holm-Sidak’s multiple comparisons test). Interestingly, in TE-671 cells, which express the fetal muscle-type nAChR, the second addition of 1 µM, ACh was also effectively desensitized at 10 and 100 µM concentrations of nicotine (Figure 4B) (*p* < 0.05, two-way ANOVA, Holm-Sidak’s multiple comparisons test). 

The piperidinyl-pyridine anabasine, acted as an agonist in both cell lines during its first addition (Figure 5A,B). As the concentration of anabasine was increased to 100 µM the response to ACh was desensitized in SH-SY5Y cells (*p* < 0.05, two-way ANOVA, Holm-Sidak’s multiple comparisons test). In comparison to the neuronal cell line, in the muscle cell line, anabasine was more effective at desensitizing the TE-671 cell response at 10 and 100 µM concentrations (*p* < 0.05, two-way ANOVA, Holm-Sidak’s multiple comparisons test). 

The 1,2-dehydropiperidine anabaseine (Figure 1) was a potent nAChR agonist in both cell lines (Figure 6A,B) but ~20 times more potent on the fetal nAChR (Table 1). In SH-SY5Y cells, the 10 µM ACh response was desensitized at 10 and 100 µM anabaseine (*p* < 0.05, two-way ANOVA, Holm-Sidak’s multiple comparisons test). In TE-671 cells the 1.0 µM ACh response was desensitized at 1, 10, and 100 µM anabaseine (*p* < 0.05, two-way ANOVA, Holm-Sidak’s multiple comparisons test).

**Figure 7:**
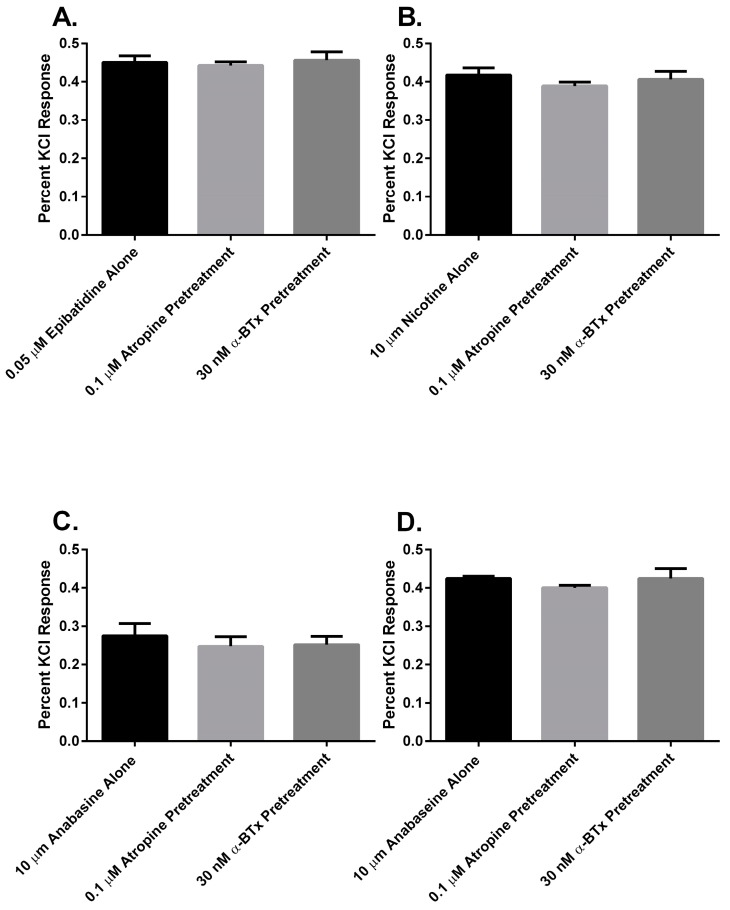
The response of SH-SY5Y cells to alkaloid agonists in the presence or absence of either 0.1 µM atropine or 30 nM α-BTx. The membrane potential sensing dye fluorescence resulting from the addition of 0.05 µM epibatidine (**A**); 10 µM nicotine (**B**); 10 µM anabasine (**C**); or 10 µM anabaseine (**D**) was measured and displayed as a percentage of the KCl response. Each column represents the results of experiments with four pairs of duplicate wells in four 96 well plates (*n* = 4).

Finally, to determine if there was a muscarinic cholinergic receptor (mAChR) or an α_7_ nAChR component to the membrane potential sensing dye fluorescence of the SH-SY5Y cells in response to alkaloid agonist addition, the cells were pretreated with either atropine or α-bungarotoxin (α-BTx) respectively (Figure 7). There were no significant differences between the membrane potential sensing dye fluorescence of the cells exposed to agonist alone or those pretreated with either of the receptor antagonists (*p* > 0.05, ANOVA).

## 3. Discussion

In this research, we found that all of the pyridine alkaloids were full agonists at the autonomic and fetal muscle nAChRs and also desensitized these receptors at concentrations that were usually less than was required for activation. The rank order for activation at the major autonomic (SH-SY5Y cell) nAChR was: epibatidine > nicotine > anabaseine > anabasine. Moreover, the responses of the cells to the agonists was likely due to the activation of autonomic receptors because the responses were insensitive to atropine, which blocks mAChR, or α-BTx, which block α_7_nAChR (Figure 7). This was also the rank order for its rapid desensitization. At the fetal (TE-671 cell) muscle nAChR the rank order for agonist potency was also the same, but there were quantitative differences: while the muscle nAChR potencies of epibatidine and nicotine were reduced and the potency of anabasine was essentially unchanged, the agonist potency of anabaseine was approximately 20 times higher for the fetal nAChR. 

We included nicotine and epibatidine in this research to serve as mechanistic reference compounds due to the large volume of research that has been done on them. Nicotine and epibatidine interactions with the muscle-type nAChR have been extensively investigated by the Dougherty-Lester Caltech group [11,18]. They have identified intermolecular interactions that result in differences in affinity and potency of cholinergic agonists at muscle-type and certain neuronal nAChRs. The amino acid tryptophan at position 149 (also known as TrpB) of the alpha subunit in the fetal muscle-type nAChR is important for two bonding interactions. First, its tryptophanyl ring forms a cation-π bond with ACh, whereas nicotine does not form a cation-π bond with TrpB at this particular nAChR. Conversely, at certain neuronal nAChRs, both nicotine and ACh form strong cation-π bonds with TrpB in the ligand binding site, and both act as potent agonists [7,8,9,10,11,18,19]. Outside of the central nervous system the lack of cation-π bond interactions at fetal muscle-type nAChRs by nicotine provides a putative explanation for the failure of this alkaloid to inhibit fetal movement and cause MCC-type deformities in livestock [12]. Based on these observations, we speculate that the formation of a cation-π bond with TrpB of the fetal muscle-type receptor expressed by the developing fetus in utero is necessary for potent receptor activation and desensitization, and contributes to the likelihood of MCC-type defects in grazing livestock. These molecular interactions between agonists and the ligand binding sites of the receptors contribute to agonist specific effects at nAChRs. In general, the more potent and effective the initial agonist is at activating a nAChR, the greater, more potent, and effective it is in causing desensitization of the receptor (Table 1) [7,20]. These results suggest that, based on the TE-671 cell A/D intercept concentrations, anabaseine (0.4 µM) would be more effective than anabasine (3 µM) or nicotine (4 µM) at desensitizing the fetal muscle-type nAChR and, as a result, inhibiting fetal movement through the paralysis of skeletal muscles. 

In this research we were particularly interested to determine if anabaseine would classically desensitize the ACh response more potently than anabasine. Past research at this laboratory with a mouse bioassay has documented the toxicities of anabaseine and anabasine enantiomers with IV LD_50_ values of 0.58 ± 0.05 mg/kg for anabaseine, 11 ± 1 mg/kg R-anabasine, and 16 ± 1 mg/kg for S-anabasine, suggesting that, in an acute mouse lethality model, anabaseine is more toxic than anabasine [21]. It is not known what actions anabaseine has on fetal movement in livestock species, such as cattle, sheep, and goats. We have, therefore, used cell-based assays to compare the actions of the known teratogen anabasine to anabaseine. In previous studies with SH-SY5Y, TE-671, and other cells, anabaseine was a more potent agonist than anabasine [22,23]. In the current study, anabaseine was also a more potent agonist than anabasine. As discussed above, the more potent agonists were also more effective at desensitizing nAChRs. The rank order of DC_50_ values in SH-SY5Y cells, which express autonomic-type nAChR, was epibatidine > nicotine > anabaseine > anabasine. The rank order of DC_50_ values (Table 1) in TE-671 cells, which express fetal muscle-type nAChR, was epibatidine > anabaseine > nicotine > anabasine. The rank order of desensitization was surprising to us because of our previous research with the day-40 pregnant goat model. Results from this work have shown that anabasine abolished all fetal movement at 30 and 60 min after IV dosing of pregnant goats, whereas nicotine only slightly reduced fetal movement at 30 and 60 min after IV dosing [12]. Nicotine is well known not to cause MCC-type deformities in livestock (reviewed in [1]). We can only speculate that there may be toxicokinetic differences between anabasine and nicotine in the fetal compartment that may prevent nicotine from inhibiting fetal movement. It seems likely that nicotine is more rapidly metabolized than the other compounds; apparently biotransformation studies of epibatidine, anabasine, and anabaseine have not been reported. However, our results do suggest that anabaseine would be more effective than anabasine at inhibiting fetal movement, based on its lower DC_50_ value at fetal muscle-type receptors. 

In conclusion, we show that all four pyridine alkaloids can classically desensitize nAChRs expressed by TE-671 and SH-SY5Y cells. Among the two alkaloids also containing a piperidine ring, anabaseine was more effective than anabasine, which we hypothesize is due to the presence of the imine double bond in the piperidinyl ring of anabaseine. Further research is needed to determine the effectiveness of other piperidine and pyridine alkaloids found in *Lupinus*, *Conium*, and *Nicotiana* spp. at desensitizing peripheral-type nAChRs and to determine how those alkaloids play a role in the formation of fetal defects in livestock species.

## 4. Conclusions

All four pyridine alkaloids were full agonists and classical desensitizers of nAChRs expressed by TE-671 and SH-SY5Y cells. Of the two piperidine-ring containing pyridine alkaloids examined, the 1,2-dehydropiperidine alkaloid anabaseine was much more potent in activating and classically desensitizing nAChRs than anabasine. We hypothesize that the presence of an imine double bond in the piperidinyl ring of anabaseine enhances binding with both types of nAChRs, particularly the muscle type. Further research is needed to determine the effectiveness of other piperidine and pyridine alkaloids found in plants, such as *Lupinus*, *Conium*, and *Nicotiana* spp., at desensitizing nAChR and to determine how those alkaloids play a role in the formation of fetal defects in livestock species.

## 5. Materials and Methods

Epibatidine was purchased from Tocris Bioscience (Bristol, UK); acetylcholine chloride, atropine, and α-BTx were purchased from Sigma-Aldrich (St Louis, MO, USA); and (−)-nicotine ditartrate from EMD Biosciences (La Jolla, CA, USA). Racemic anabasine was isolated from tree tobacco (*Nicotiana glauca*) and purified in-house by Richard Keeler [22,24]. Anabaseine dihydrochloride was synthesized by Dr. Ferenc Soti (Kem laboratory, University of Florida, Gainesville, FL, USA). Fetal bovine serum was purchased from HyClone Laboratories (Logan, UT, USA); penicillin/streptomycin and Dulbecco’s Modified Eagle’s Medium from Life Technologies (Grand Island, NY, USA); and trypsin-ethylenediaminetetraaacetic acid (EDTA) from American Type Culture Collection (ATCC) (Manassas, VA, USA). Membrane Potential R8034 blue dye kits were obtained from Molecular Devices (Sunnyvale, CA, USA). The SHSY-5Y and TE-671 cell lines were obtained from ATCC (Manassas, VA, USA).

### 5.1. Cell Culture

Cells were grown and maintained in supplemented filter-sterilized growth medium (Dulbecco’s Modified Eagle’s Medium, 10% fetal bovine serum and 1% penicillin/streptomycin) at 37 °C, 5% CO_2_, in a humidified incubator. Twenty-four hours prior to membrane potential sensing dye fluorescence assays, cells were transferred to 96-well black-walled, clear-bottom assay plates obtained from Corning Incorporated (Corning, NY, USA). Molecular Devices blue dye was dissolved in HBSS-HEPEs (20 mM) at concentration of 1 vial: 360 mL HBSS-HEPES. Both cells and dye were brought to room temperature and the dye loaded into the cell assay plates 30 min prior to measuring changes in fluorescence by a Flexstation III (Molecular Devices Corporation, Sunnyvale, CA, USA) as previously described [22].

### 5.2. Activation and Desensitization Experiments

Desensitization is defined as a receptor state that is unresponsive to continued agonist exposure and was first quantitatively described in 1957 by Katz and Thesleff [25]. In this research, a statistically significant decrease (*p* < 0.05, as denoted by an asterisk in the figures) in the membrane potential sensing dye fluorescence after the second addition of agonist was used as a measure of receptor desensitization. The desensitization assays presented in this research are based upon the work of Campling et al. [26] and Knapman et al. [20]. In our experiments, we used a dual-agonist addition protocol to desensitize nAChR (Figure 1B). A baseline fluorescent response was measured for 18 s, then 25 µL of agonist in dye-saline solution was added, and the membrane potential sensing dye fluorescence measured. The membrane potential sensing dye fluorescence was then allowed to stabilize before a second 25 µL addition of agonist; in this case, a maximally stimulating ACh containing saline at 98 s (10 µM ACh for SH-SY5Y cells and 1 µM ACh for TE-671 cells). The ACh concentration was based on concentration-effect experiments that identified the lowest concentration of ACh that provided a maximum membrane potential sensing dye fluorescence in each cell line (Figure S1). At 180 s into the experiment, 25 µL of KCl, in dye-saline solution, was added to attain a final concentration of 40 mM KCl for complete depolarization of the cells. Fluorescent readings were taken every 1.12 s for 255 s with a total of 228 readings per well using a Flexstation III with wavelength excitation at 530 nm, emission of 565 nm, and a cut off wavelength of 550 nm. Compound responses were calculated as previously described [21]. For experiments with atropine and α-BTx, the SH-SY5Y cells were prepared as above and then manually exposed to the antagonists just prior to placing them onto the Flexstation for agonist addition and fluorescence detection.

### 5.3. Statistical Analysis

The membrane potential sensing dye fluorescence of the cells was normalized to the maximum response generated by each agonist in each concentration effect curve with Prism version 6.03 (GraphPad Software, San Diego, CA, USA, 2015), except for Figure 2, where the responses were normalized to epibatidine, as previously described [8] to allow for a visual comparison of alkaloid agonism. Fifty percent effective concentration (EC_50_) and fifty percent desensitization (DC_50_) values were calculated using nonlinear regression analysis in Prism, as previously described, using the log (agonist) versus a normalized response-variable slope equation. To calculate the agonist concentration of the mid-point of the “smoldering activation” concentration range [26], the percent response of the A/D intercept was estimated by drawing a line from the intercept to the *y*-axis and rounding the response to the nearest whole percent. That percent response value, the EC_50_ concentration, and the hill slope were used with the EC_anything_ calculator from GraphPad software [27] to estimate the A/D intercept presented in Table 1. A two-way ANOVA was used to detect differences between multiple means using a Holm-Sidak’s multiple comparisons test which was corrected for multiple comparisons. A one-way ANOVA was used to analyze cell response to the presence or absence of antagonists. In all cases, the limit for statistical significance was set at *p* < 0.05. 

## Figures and Tables

**Table 1:** Calculated EC_50_ and DC_50_ estimates of compounds in TE-671 and SH-SY5Y cells.

Compound	EC_50_, µM (CI ^a^)	SH-SY5Y Cells DC_50_ µM (CI ^a^)	A/D ^b^ Intercept µM	EC_50_ µM (CI ^a^)	TE-671 Cells DC_50_ µM (CI ^a^)	A/D ^b^ Intercept µM
Epibatidine	0.018	0.015	0.02	0.16	0.3	0.3
	(0.009–0.04)	(0.006–0.04) ^a^		(0.14–0.2)	(0.1–0.6)	
Nicotine	3.4	1.0	0.9	9.5	1.9	4
	(1.4–5.1)	(0.4–2.7)		(7.1–13)	(0.3–12)	
Anabasine	15.3	5.6	8	16.7	2.3	3
	(6.7–67)	(1.7–19)		(13–21)	(0.7–7.5)	
Anabaseine	5.9	2.4	2	0.31	0.6	0.4
	(4.4–36)	(1.0–5.8)		(0.2–0.49)	(0.2–2.1)	

^a^ Ninety-five percent confidence interval; ^b^ Intercept between the agonist concentration-effect curve and the ACh desensitization curve.

## Supplementary Materials

The following are available online at www.mdpi.com/2072-6651/8/7/204/s1, Figure S1: The concentration-response relationships for ACh using membrane potential sensing dye fluorescence in SH-SY5Y (**A**) and TE-671 (**B**) cells. The cellular responses to agonist were n*o*rmalized to the maximum epibatidine response for each cell line.
