# Supplementary material for: Activation and Desensitization of Peripheral Muscle and Neuronal Nicotinic Acetylcholine Receptors by Selected, Naturally-Occurring Pyridine Alkaloids
Source: Toxins (Basel). 2016 Jul 4;8(7):204. doi: 10.3390/toxins8070204 (PMC4963837; doi:10.3390/toxins8070204)
Supplement: Supplementary file 1 [file toxins-08-00204-s001.pdf]

Supplementary Materials: Activation and Desensitization of Peripheral Muscle and Neuronal Nicotinic Acetylcholine Receptors by Selected, Naturally-Occurring Pyridine Alkaloids

Benedict T. Green , Stephen T. Lee , Kevin D. Welch , Daniel Cook and William R. Kem

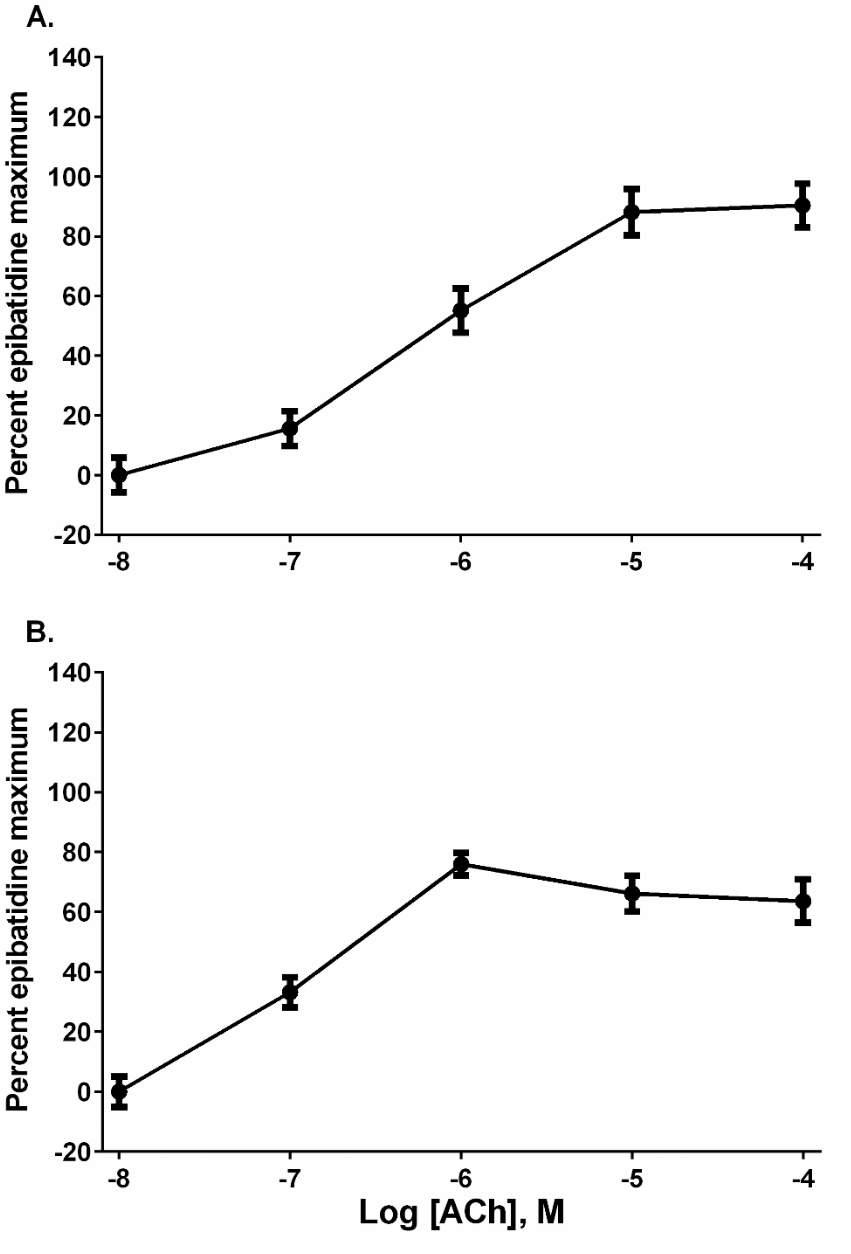

Figure S1. The concentration-response relationships for ACh using membrane potential sensing dye fluorescence in SH-SY5Y (**A**) and TE-671 (**B**) cells. The cellular responses to agonist were normalized to the maximum epibatidine response for each cell line.
